# The Effects of Acute Physical Exercise on Memory, Peripheral BDNF, and Cortisol in Young Adults

**DOI:** 10.1155/2016/6860573

**Published:** 2016-06-29

**Authors:** Kirsten Hötting, Nadine Schickert, Jochen Kaiser, Brigitte Röder, Maren Schmidt-Kassow

**Affiliations:** ^1^Biological Psychology and Neuropsychology, University of Hamburg, Von-Melle-Park 11, 20146 Hamburg, Germany; ^2^Institute of Medical Psychology, Goethe University Frankfurt, Heinrich-Hoffmann-Strasse 10, 60528 Frankfurt am Main, Germany

## Abstract

In animals, physical activity has been shown to induce functional and structural changes especially in the hippocampus and to improve memory, probably by upregulating the release of neurotrophic factors. In humans, results on the effect of acute exercise on memory are inconsistent so far. Therefore, the aim of the present study was to assess the effects of a single bout of physical exercise on memory consolidation and the underlying neuroendocrinological mechanisms in young adults. Participants encoded a list of German-Polish vocabulary before exercising for 30 minutes with either high intensity or low intensity or before a relaxing phase. Retention of the vocabulary was assessed 20 minutes after the intervention as well as 24 hours later. Serum BDNF and salivary cortisol were measured at baseline, after learning, and after the intervention. The high-intensity exercise group showed an increase in BDNF and cortisol after exercising compared to baseline. Exercise after learning did not enhance the absolute number of recalled words. Participants of the high-intensity exercise group, however, forgot less vocabulary than the relaxing group 24 hours after learning. There was no robust relationship between memory scores and the increase in BDNF and cortisol, respectively, suggesting that further parameters have to be taken into account to explain the effects of exercise on memory in humans.

## 1. Introduction

Physical exercise has beneficial effects on neuroplasticity and cognition [1]. The largest and most reliable effects have been reported for executive functions [2]. Recent studies have suggested that memory might benefit from physical exercise as well [3–6]. These behavioral results are in accord with neuroanatomical observations showing that the volume of the human hippocampus, a key structure for the consolidation of long-term memories, increased in humans who had exercised for one year [7, 8].

Not only chronic effects of exercise interventions lasting for months up to years have been reported, but also a single bout of exercise has been shown to increase performance on a large variety of cognitive tasks [9, 10]. Compared to other cognitive domains, the number of studies on the effects of a single bout of exercise on memory is rather limited and their results are inconsistent so far with some studies showing beneficial effects, no effects, or even detrimental effects. The results seem to depend on the exercise intensity, the type of memory being tested, and the timing of exercise relative to the memory task [11].

Models of memory consolidation emphasize the dynamic nature of memory representations by proposing two main memory stages: a label state in which memories are susceptible to enhancements or improvements and a stable state in which they are rather insensitive to any treatment [12–15]. The transient label state is seen shortly after learning and after the reactivation of memory traces. Thus, memory might especially be modified by physical exercise when performed during early phases of memory consolidation. In most studies reporting beneficial effects of acute physical exercise on long-term memory, however, participants exercised either before or during learning [16–20]. Based on the results of these studies it is not possible to distinguish whether exercising facilitates memory encoding, consolidation, or both processes.

The results of studies in which participants exercised after learning were mixed. For example, Labban and Etnier [16] did not find a significant memory benefit in participants exercising after a learning session compared to a resting condition, while memory improved in participants who had exercised before learning. This finding contrasts with results of McNerney and Radvansky [21] and Segal et al. [22] who showed that aerobic exercise immediately after encoding enhanced memory. These studies differed with regard to the duration of the exercise intervention, the type of memory being tested, and the delay between exercising and recall. While participants in the study by Segal et al. [22] exercised for only six minutes and had to recall their memories 60 minutes later, participants in the study by Labban and Etnier [16] exercised for 30 minutes and were asked to recall their memories immediately after exercise. It might be speculated that the high arousal induced by the exercise intervention might have impaired retrieval in the study of Labban and Etnier [16]. McNerney and Radvansky [21], however, reported a positive effect of a short bout of high intense exercise after learning on immediate tests of procedural and declarative memory. The superior performance of the exercise group compared to a control group sustained over a one-week delay. This is in line with a further report on the effects of exercise on procedural memories [23]. In this study, participants learned a visuomotor task either before exercising for 15 minutes, after exercising, or after the same time of relaxing. The physical exercise did not affect the acquisition of the task nor the retention one hour after the intervention. However, participants who exercised after the learning task showed better long-term retention seven days later compared to participants who exercised before learning. The authors concluded that positive effects of exercise might be maximized when performed during early stages of memory consolidation.

In sum, results so far suggest that the timing of exercise relative to memory encoding or retrieval as well as exercise intensity might influence the effects of acute exercise on memory [11]. However, only a few studies have applied exercise interventions after learning new material and, to our knowledge, none of these studies has varied exercise intensity.

The brain-derived neurotrophic factor (BDNF) and cortisol have been discussed as possible neuroendocrinological mediators of exercise induced memory changes. BDNF has been recognized as an important candidate molecule for exercise induced plasticity [24]. BDNF is involved in the cellular and subcellular mechanisms of learning and memory, for instance, in the induction and maintenance of long-term potentiation and in structural remodeling of dendritic spines and neurogenesis [25–27]. Exercise has been shown to increase the expression of BDNF in the hippocampus and perirhinal cortex, which correlated with better learning and memory [28, 29]. BDNF is synthesized both in the central nervous system and in somatic cells outside the CNS [24]. Central BDNF, however, cannot be measured in living humans. Therefore, changes in peripheral blood serum or plasma levels have been used as an estimate of central BDNF in humans. There are results suggesting that BDNF crosses the blood-brain barrier bidirectionally [30]. Correlations between BDNF levels in the periphery and the brain have been reported in animals [30, 31]; however, other results questioned a direct association between central and peripheral BDNF responses [32]. Acute physical exercise induces a reliable transient increase in peripheral BDNF [20, 33–40]. There is evidence that this effect depends on the exercise intensity with an increase in BDNF only after high-intensity exercise protocols [19, 34, 37, 39].

Exercise can be considered as a physical stressor, which activates the hypothalamic-pituitary-adrenal axis [41]. Accordingly, an elevation of cortisol has been observed after acute exercise with at least moderate intensity [42], but not after light exercise [19]. It is known that glucocorticoids enhance memory consolidation; however, depending on the timing of the stressor, a stress-related increase in glucocorticoids might impair memory retrieval and reconsolidation [43]. Thus, stress hormones released during exercise might mediate exercise related memory effects.

The aim of the present study was to assess the effects of a single bout of physical exercise on memory consolidation and the underlying neuroendocrinological mechanisms. Participants learned new vocabulary before exercising for 30 minutes with either high intensity or low intensity or before a relaxing phase. Vocabulary learning was chosen as memory task because of its high ecological validity. Moreover, high impact exercise has been shown to quicken vocabulary learning [20]. We expected a dose-dependent effect of physical exercise on memory consolidation with better retention of the newly learned vocabulary with increasing exercise intensity. Based on previous reports of BDNF and cortisol enhancements, especially after intense exercise protocols, increases in BDNF and cortisol were expected for the high-intensity group. Moreover, we hypothesized a direct relation between changes in BDNF and cortisol and memory consolidation.

## 2. Materials and Methods

### 2.1. Participants

Eighty-one young and healthy university students participated in the experimental session (40 female, 41 male, mean age: 22 years, SD: 2.36, age range 18–29 years, mean BMI: 21.7 kg/m^2^, SD: 1.85). All participants were right-handed as determined by the Edinburgh handedness inventory [44]. Exclusion criteria were a history of psychiatric or neurological disorders, cardiovascular diseases, smoking, medication (except contraceptives), obesity, competitive sports, and any knowledge of Polish or other Slavic languages (since participants were asked to learn Polish vocabulary). All participants were native German speakers.

### 2.2. Ethics Statement

The Ethics Committee of the Medical Faculty of Goethe University Frankfurt approved the study. It was conducted in accordance with the principles laid down in the Declaration of Helsinki (2013). All participants were informed about the aims of the study and gave written consent.

### 2.3. Procedure

Participants were screened in a preexperimental session. The second session comprised the experimental learning phase and the exercise intervention. During this experimental session, three blood samples were collected for the BNDF analysis and four saliva samples for the cortisol analysis. Moreover, participants filled in an online test of vocabulary retention 24 hours after the experimental session.

#### 2.3.1. Preexperimental Screening

During the preexperimental screening session participants completed several questionnaires to assess demographic variables, their physical activity level, and medical contraindications for a cardiovascular fitness test and physical exercise. The physical activity level was measured with the Freiburg Questionnaire of Physical Activity (FQPA) [45]. Furthermore, participants had to indicate the number of foreign languages they had learned and the number of musical instruments they played as foreign language processing and musical expertise are known to interact [46–48]. We assessed the state of health during the last five years using a short questionnaire asking for specific disorders, such as vertigo, impaired vision, chest pain, tachycardia, or dyspnea, which could potentially result from cardiovascular diseases. We also checked for other chronic diseases such as diabetes, asthma, epilepsy, respiratory disorders, and disorders of the musculoskeletal system, as well as surgical interventions within the last five years, pregnancy, and acute infections.

During the screening session, participants took part in a vocabulary test to assess their baseline learning abilities. They listened to 40 pseudowords each followed by a legal German word. After a delay period of 20 minutes, only the pseudowords were presented auditorily and participants had to write down the associated German words. To conform with our previous studies [17–19], participants who were able to memorize more than 20 pseudowords were excluded from the main experiment. Sixteen participants were excluded due to this criterion. Fifteen participants were not considered due to health problems. Finally, five participants were rejected since they were bilinguals and one due to smoking.

To assess the participants' cardiovascular fitness and to determine an individually adjusted intensity for the exercise intervention, they performed an incremental exercise test on a cycle ergometer (Conditronic 100 PV/ZR-NS, Dynavit, Germany). Cycling started at 30 Watts and was increased in 30-Watt steps [49]. Each step took three minutes. The cadence had to be at least 60 rotations per minute (RPM). Heart rate was measured continuously with a chest strap (Polar S810, Polar, Büttelborn, Germany). At the end of each step, participants had to estimate their perceived exertion by using the Borg scale. This scale has previously been shown to be a reliable and valid tool for measuring perceived exertion [50]. The exercise test was terminated when participants reported subjective exhaustion or when their cadence was below 60 RPM for more than 10 sec. VO_2max_ was estimated as described in [51]. At the beginning of the study, all participants were instructed to avoid changes in their daily life activity, in particular, their physical activity, until the second vocabulary test had been completed at the day after the main experiment.

Participants were pseudorandomly assigned to one of the three experimental groups, a high-intensity physical exercise group (*N* = 26), a low-intensity physical exercise group (*N* = 27), and a relaxing group (*N* = 28), matched according to gender, maximum Watt rate in the incremental exercise test, and their performance in the pseudoword learning task during the screening session. That is, we formed triplets which were controlled for the above-mentioned parameters and assigned them randomly to the respective experimental groups.

#### 2.3.2. Experimental Session

**Figure 1:**
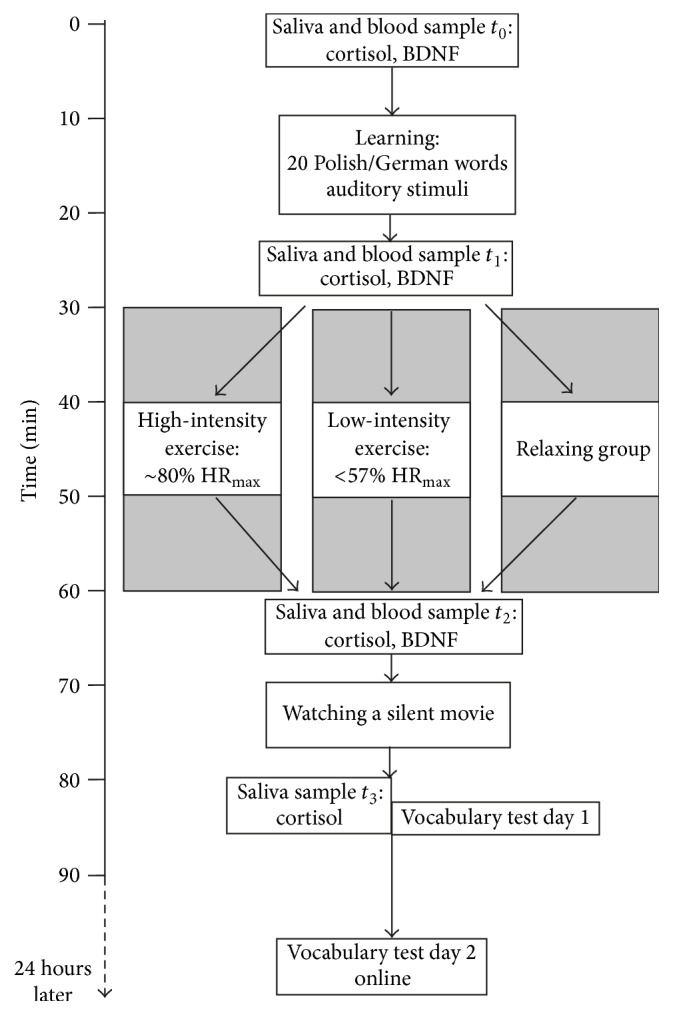
Overview of the experimental learning session with the timing of blood and saliva collection, learning, exercise intervention, and vocabulary tests.

An overview of the experimental procedure with the timing of the learning phase, memory tests, exercise intervention, and the blood and saliva collections is depicted in Figure 1.

The experimental session started between 2:30 pm and 5:00 pm. During the learning phase, participants listened to 20 Polish-German word pairs (10 nouns and 10 verbs) presented via headphones (Philips SHP 1900) while sitting on a desk chair. Participants were told that their memory for the vocabulary would be tested on the same day and the day after. Two blocks were run with each Polish-German pair presented once in each block. The order of vocabulary pairs was randomized for each block and each participant. Initially, all stimuli were normalized to an intensity level of 75 dB(A). At the beginning of the experimental session, participants were allowed to adjust the loudness level to their individual preference. It was kept constant across the learning session. Polish and German items were spoken by two females, native speakers of Polish and German, respectively. The stimulus onset asynchrony (SOA) of Polish-German vocabulary pairs was 2 sec within a vocabulary pair and 6 sec between successive vocabulary pairs. During this pause, participants were asked to repeat the just heard vocabulary pair aloud.

After they encoded the Polish-German vocabulary, participants of the physical exercise groups were asked to exercise on a cycling ergometer (Conditronic 100 PV/ZR-NS, Dynavit, Germany) for 30 minutes. The heart rate was constantly monitored with a chest strap (Polar S810, Polar, Büttelborn, Germany). For the low-intensity group, the intensity was set to <57% of their individual maximal heart rate (HR max) as determined during the exercise test (see preexperimental screening session), while it was set to 80% of their HR max ± 5 beats/min for the high-intensity group. This corresponded to “very light” and “vigorous” physical exercise according to [52]. During the initial five minutes of the physical intervention, resistance was increased until participants' heart rate reached their prescribed target heart rate. Additionally, participants had to indicate their perceived exertion level on the Borg scale after 15 min and 30 minutes, respectively. Members of the relaxing group were resting in a canvas chair for thirty minutes after they had learned the new vocabulary.

After the intervention, all participants watched a silent movie called “Shaun the Sheep-Abracadabra” for 20 minutes. During this period arousal decreased in the exercise groups. The participants' memory for the newly learned vocabulary was tested thereafter. They listened to 40 Polish words (20 old items and 20 new items) and had to respond, if possible, with the German translation using a standard computer keyboard. The number of correctly recalled old words was used as memory score. The reason for adding new items in the recall phase is that we have planned to measure additional EEG during the recall phase in a follow-up experiment to study the old/new effect. Response time for the vocabulary test was limited to 8 s for each item.

At the end of the experimental session, all participants were asked to indicate their motivation and perceived difficulty to learn the new vocabulary, their perceived exertion during exercising, their current daily stress level and workload at the university, and the quality of their last night's sleep. Answers were given on a 5-point Likert scale ranging from ‘‘low” to ‘‘high.” Furthermore, we asked the participants about their caffeine and alcohol consumption and the hours they slept during the last 24 hours. Water was provided to all participants throughout the session.

Twenty-four hours after the learning session participants took part in an additional customized online vocabulary test. They were able to access the test from their computer at home. The access was temporally limited to make sure that participants adhered to their individual test time (±1 hour). Participants were asked to listen again to the Polish words encoded during the experimental learning session and were asked to enter the correct German counterpart.

### 2.4. Blood Sampling and Analysis of BDNF Serum Concentrations

For BDNF analysis, 4.5 mL of venous blood from the antecubital vein was collected with a clotted blood tube at three time points. The first blood sample was taken at the beginning of the experimental session. The second blood sample was collected immediately after the learning phase and the third one was taken immediately after the intervention (exercising or relaxing).

All samples clotted within 30 minutes at a temperature of 21°C. After the clotting period, samples were centrifuged for 10 minutes with 4800 rounds per minute using the Heraeus Labofuge 200 (Thermo Fisher Scientific, Germany). Serum was immediately pipetted into separate SafeSeal microtubes (Sarstedt, Nürnberg, Germany). Samples were stored at −23°C for a mean of 13.5 days and then transferred to a −80°C freezer until the analysis started. BDNF levels in serum were measured using the Quantikine® Human BDNF Immunoassay from R&D Systems (Wiesbaden, Germany) with intra- and interassay coefficients of variation in the range between 8.8 and 11.4 for values between 7.24 and 41.6 ng/mL. The minimum detectable BDNF dose was less than 20 pg/mL, according to the manufacturer's information. BDNF analysis was performed by the Institute of Laboratory Medicine, Clinical Chemistry and Molecular Diagnostics in Leipzig.

### 2.5. Saliva Sampling and Analysis of Cortisol Concentrations

Saliva samples for the cortisol analysis were taken at four time points. The first three samples were collected immediately before the blood sampling, that is, at the beginning of the experimental session, after the learning phase, and after exercising/relaxing. The fourth saliva sample was taken 20 minutes after exercising, that is, after participants had watched the silent movie.

The “Salivette” (Sarstedt AG & Co., Nümbrecht) collection devices were used for collecting saliva. They consist of a cotton swab in a suspended insert which itself is placed in a centrifuge vessel. Participants were instructed to gently chew on the cotton swabs for at least one minute. Afterwards they return the saturated swab to the suspended insert without touching it. To reduce errors, participants were not allowed to eat or drink anything else than water for half an hour before the testing session started and during the session. Salivary samples were stored at −23°C until the biochemical analysis was conducted, which was performed by the Dresden Lab Service GmbH. Saliva samples were maximally stored for 19 days. Samples were analyzed according to the protocol described in Dressendörfer et al. [53]. The concentration of free salivary cortisol was analyzed using a luminescence immunoassay (IBL, Hamburg, Germany) with intra- and interassay precision of 4.5% and 4.3%, respectively.

### 2.6. Data Analysis

Changes in memory scores, BDNF, and cortisol after the exercise intervention were compared between experimental groups by a univariate analysis of variance (ANOVA) with the factors Group (high-intensity exercise versus low-intensity exercise versus relaxing) and Time (day 1 versus day 2 for the memory score, *t*_0_, *t*_1_, and *t*_2_ for BDNF, and *t*_0_, *t*_1_, *t*_2_, and *t*_3_ for cortisol).

As we had a specific hypothesis about the direction of the exercise effect, differences between groups were further analyzed with planned contrasts or a linear trend analysis whenever the main effect of Group or the Group × Time interaction was at least marginally significant (*p* < 0.1). In the contrast analyses, results of the relaxing group were compared to those of the high-intensity group (first contrast) and to those of the low-intensity exercise group (second contrast). For the trend analysis, we expected a linear trend with increasing scores of the dependent variables with increasing exercise intensity (−1: relaxing, 0: low intensity, and 1: high intensity).

Possible correlations between vocabulary retention (vocabulary score day 2 minus day 1) and changes in BDNF (*t*_2_ minus *t*_0_) and Cortisol (*t*_2_ minus *t*_0_, *t*_3_ minus *t*_0_) after exercising/relaxing were explored by nonparametric correlations using Kendall's Tau because the data were not normally distributed.

## 3. Results

### 3.1. Demographics and Manipulation Check

Groups were pseudorandomly assigned to a high-intensity exercise group, a low-intensity exercise group, or a relaxing group. Groups did not differ with regard to age, sex, body mass index, daily physical activities, physical fitness, the number of foreign languages learned, number of participants playing a musical instrument, or memory scores during preexperimental screening (see Table 1). The number of women taking oral contraceptives differed between groups, *X* ^2^(2)   = 7.10, *p* = 0.029, and was highest in the high-intensity exercise group. As contraceptives might influence memory performance and neuroendocrinological parameters [54–56], we ran additional analyses excluding women without contraceptives. The pattern of results remained the same as in the complete sample suggesting that differences in the number of women taking contraceptives did not account for the group differences reported in the following (see Supplementary Material available online at http://dx.doi.org/10.1155/2016/6860573). The number of women not taking contraceptives was too small in this sample to analyze their data separately or to analyze the interaction between the use of contraceptives and exercise.

As expected, the heart rate during the experimental intervention differed between groups, *F*(2,75) = 533.18, *p* < 0.001, with a significantly higher heart rate for the exercise groups compared to the relaxing group (planned contrast *t*(75) = 27.00, *p* < 0.001) and a significant difference between the high-intensity group and the low-intensity group (planned contrast *t*(75) = 18.08, *p* < 0.001). The latter additionally differed in the rating of the perceived exhaustion after exercising, *t*(51) = 13.03, *p* < 0.001.

### 3.2. Memory for Newly Learned Vocabulary

One participant assigned to the relaxing group was excluded from the analyses of memory scores, as he did not recall any word correctly, neither at day one nor at day two.

**Figure 2:**
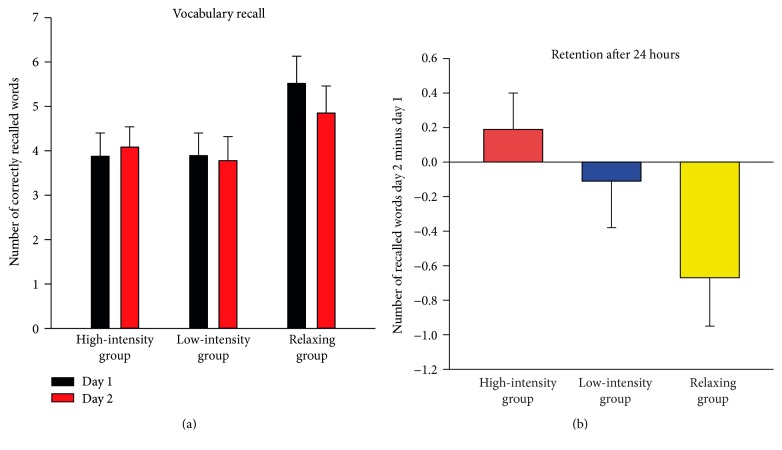
Memory for newly learned vocabulary. (a) Mean number of correctly recalled words at day one (60 minutes after learning) and at day two (24 h after learning), separately for the high-intensity group, the low-intensity group, and the relaxing group. (b) Retention after 24 hours, defined as the difference between numbers of correctly recalled words at day one minus day two. Error bars depict ±1 standard error of the mean.

Exercising after learning did not enhance the absolute number of recalled vocabulary, main effect of Group *F*(2, 77) = 1.99, *p* = 0.144, *η* ^2^ = 0.049 (Figure 2(a)). The Time × Group interaction was marginally significant, *F*(2, 77) = 2.84, *p* = 0.064, *η* ^2^ = 0.069, indicating that group differences in vocabulary retention depended on the time of testing. The contrast analysis for the vocabulary score at day one revealed that the relaxing group initially recalled more words than both the high-intensity exercise group, *t*(77) = −2.10, *p* = 0.039, and the low-intensity exercise group, *t*(77) = −2.12, *p* = 0.038. Planned contrasts for day two did not reveal any significant difference between the relaxing group and the exercise groups, all *p* > 0.16.

To explore the changes in vocabulary retention after 24 hours, the difference score “recalled words day two” minus “recalled words day one” was compared between the three groups (Figure 2(b)). There was a significant linear trend, *F*(1, 77) = 5.51, *p* = 0.022, indicating that vocabulary retention increased proportionately with increasing exercise level. Moreover, planned contrasts for these difference scores showed a significant lower loss of memory for the high-intensity exercise group compared to the relaxing group, *t*(77) = 2.35, *p* = 0.022.

### 3.3. Brain-Derived Neurotrophic Factor (BDNF)

Blood samples to assess BDNF serum level were taken at baseline (*t*_0_), after learning (*t*_1_), and after exercising/relaxing (*t*_2_), respectively. The venous puncture failed in two participants. Furthermore, data of two participants with BDNF values more than three standard deviations above the sample mean at at least one time point were excluded. Thus, the BDNF analyses were based on *N* = 77 participants (*N* = 26 high-intensity exercise group, *N* = 23 low-intensity exercise group, and *N* = 28 relaxing group).

**Figure 3:**
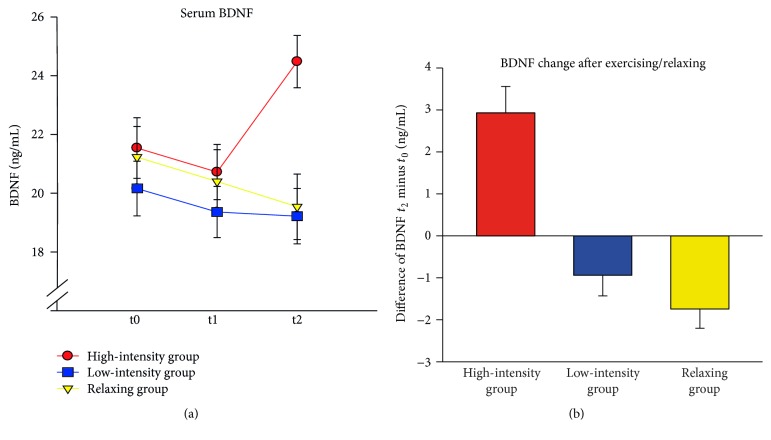
(a) Mean serum BDNF at baseline (*t*_0_), after learning (*t*_1_), and after exercising/relaxing (*t*_2_) separately for the high-intensity exercise group, the low-intensity exercise group, and the relaxing group. (b) Mean changes in BDNF from baseline to the assessment after exercising/relaxing. Positive values indicate an increase in BDNF. Error bars depict ±1 standard error of the mean.

Serum BDNF only increased after exercising in the high-intensity exercise group, Time × Group *F*(4,148) = 17.99, *p* < 0.001, *η* ^2^ = 0.327 (Figure 3(a)). The analysis per time point showed that groups did not differ with respect to BDNF at *t*_0_ and at *t*_1_ (all *F* < 0.50, all *p* > 0.60) but that there was a significant effect of Group after exercising at *t*_2_, *F*(2,74) = 8.68, *p* < 0.001. Planned contrasts for *t*_2_ confirmed a significant difference between the high-intensity exercise group and the relaxing group, *t*(74) = 3.58, *p* = 0.001, while the low-intensity exercise group and the relaxing group did not differ, *t*(74) = −0.22, *p* = 0.824. When comparing the change from baseline to postintervention (*t*_2_ minus *t*_0_), the linear trend indicating an increase in BDNF with increasing exercise intensity was significant, *F*(1,74) = 40.08, *p* < 0.001. As seen in Figure 3(b), this effect was mainly driven by the large increase in the high-intensity exercise group.

### 3.4. Cortisol

Saliva samples to assess cortisol levels were taken at baseline (*t*_0_), after learning (*t*_1_), immediately after exercising/relaxing (*t*_2_), respectively, and again 20 minutes after *t*_2_ (*t*_3_). Cortisol data of two participants were missing due to sampling errors. Furthermore, five participants with cortisol levels more than three standard deviations above the sample mean at at least one time point were excluded from the analysis. Thus, cortisol analyses were based on *N* = 74 participants (*N* = 26 high-intensity exercise group, *N* = 23 low-intensity exercise group, and *N* = 25 relaxing group).

**Figure 4:**
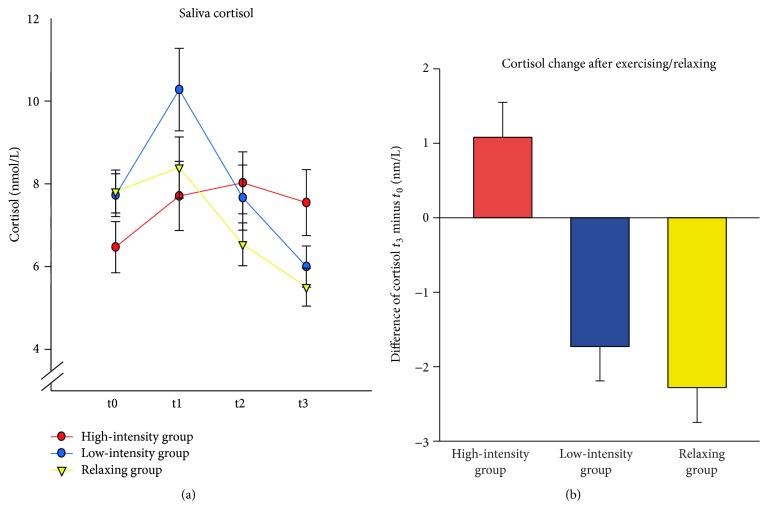
(a) Mean saliva cortisol at baseline (*t*_0_), after learning (*t*_1_), after exercising/relaxing (*t*_2_), and 20 minutes after exercising (*t*_3_), separately for the high-intensity exercise group, the low-intensity exercise group, and the relaxing group. (b) Mean changes in cortisol from baseline to the assessment 20 minutes after exercising/relaxing. Positive values indicate an increase in cortisol. Error bars depict ±1 standard error of the mean.

Cortisol levels changed differentially across time for the three experimental groups, Time × Group *F*(6,213) = 6.53, *p* < 0.001, *η* ^2^ = 0.155 (Figure 4(a)). Comparisons between groups for each time point showed no significant group differences at *t*_0_, *t*_1_, and *t*_2_ (all *F* < 2.35, all *p* > 0.10). Twenty minutes after exercising (*t*_3_), however, there was a significant main effect of Group *F*(2,71) = 3.16,  *p* = 0.049, with the high-intensity group showing a larger cortisol level compared to the resting group (contrast analysis high-intensity group versus relaxing group *t*(71) = 2.41, *p* = 0.019; low-intense exercise group versus relaxing group *t*(71) = 0.57, *p* = 0.569). Furthermore, the change in cortisol levels from *t*_0_ to *t*_3_ increased with increasing exercise intensity (linear trend analysis for the difference score of cortisol *t*_3_ minus *t*_1_: *F*(1,71) = 15.34, *p* < 0.001, Figure 4(b)). As for BDNF, however, this was mainly driven by the increase in the high-intensity group.

### 3.5. Correlations between Vocabulary Retention, BDNF, and Cortisol

When analyzing data of all participants, there was a significant positive correlation between the increase in BDNF after exercise/relaxing (BDNF *t*_2_ minus *t*_0_) and vocabulary retention (day 2 minus day 1), *τ* = 0.17, *p* = 0.046. However, this correlation was not significant when analyzing the high-intensity group only, *τ* = 0.11, *p* = 0.463. There was no positive relationship between the increase in BDNF after exercising/relaxing and the absolute number of recalled words, neither at day one nor at day two (*τ* < 0.15, *p* > 0.05).

An analysis including all participants revealed a significant positive correlation between the increase in cortisol immediately after exercise/relaxation (cortisol *t*_2_ minus *t*_0_) and vocabulary retention (day 2 minus day 1), *τ* = 0.19, *p* = 0.027, and a marginally significant positive correlation between the increase in cortisol 20 minutes after exercise/relaxation (cortisol *t*_3_ minus *t*_0_) and vocabulary retention (day 2 minus day 1), *τ* = 0.15, *p* = 0.076. There was no positive relationship between these variables when analyzing the high-intensity group only, *τ* = −0.04, *p* = 0.819 for cortisol *t*_2_ minus *t*_0_ and *τ* = −0.17, *p* = 0.271 for cortisol *t*_3_ minus *t*_0_. The increase in cortisol after exercising/relaxing did not correlate with the absolute number of recalled words (*τ* < 0.11, *p* > 0.22).

The larger the increase in BDNF immediately after exercising (BDNF *t*_2_ minus *t*_0_), the larger the increase in cortisol 20 min later (Cortisol *t*_3_ minus *t*_0_), *τ* = 0.23, *p* = 0.005. The correlation between BDNF and cortisol increase was also significant when analyzing the high-intensity exercise group only, *τ* = 0.32, *p* = 0.023.

## 4. Discussion

The aim of the present study was to assess the effects of a single bout of exercise on memory consolidation and the release of the neuroendocrinological parameters BDNF and cortisol. Exercise after learning did not enhance the recall of newly learned vocabulary at the day of learning and exercising. On the contrary, at that time point, participants of a relaxing group recalled more words than participants of a high-intensity exercise group and participants of a low-intensity exercise group. However, participants who engaged in high-intensity exercise for 30 minutes after learning showed better memory consolidation compared to the relaxing group assessed 24 hours after the learning session; that is, they retained more of the initially learned words. Moreover, high-intensity physical exercise led to a significant increase in peripheral BDNF and saliva cortisol.

In contrast to most previous studies assessing the effects of acute exercise on memory [16, 19, 20], participants in the present study did not exercise before or during the learning phase but after learning. Thus, we were able to study the effects of physical exercise during early phases of memory consolidation while holding learning conditions constant across groups. Experimental interventions to enhance (e.g., strychnine administration) or impair memories (e.g., electroconvulsive shocks, administration of protein synthesis inhibitors, and behavioral distractors) have an impact on later recall especially when administered within a short time window after learning when synaptic consolidation processes take place [12, 57]. In parallel, or as a consequence, mechanisms of system consolidation are initiated which take days up to years and are important to stabilize long-term memories [57]. Therefore, one might speculate that physical exercise enhances long-term memory by facilitating early stages of memory consolidation.

The timing of the observed behavioral effects fits well with this explanation: 20 minutes after the exercise intervention, there was no beneficial effect of exercise on memory. On the contrary, at that time point, participants of the relaxing group recalled more words than participants of the exercise groups. However, participants in the high-intensity group did not show any forgetting of the learned words after 24 hours while there was a significant decrease in the relaxing group. Thus, the immediate memory was better in the relaxing group, but the high-intensity exercise group showed better long-term consolidation of the vocabulary. The difference between the immediate memory test and the 24-hour test for the low-intensity group was between the same difference for the high-intensity group and the relaxing group, suggesting a dose-response relationship between exercise intensity and memory consolidation. The results are in line with reports of previous studies showing that exercising after learning did not improve immediate memory [16], but improvements were only seen after a delay of at least 24 hours [23]. Roig et al. [58] argued that memory tests administered too early after encoding, when memory traces are still undergoing consolidation, are not able to detect exercise induced memory gains and might even interrupt the consolidation process. As the timing of memory tests relative to the exercise interventions differ between studies, this variability might explain inconsistent results in previous studies.

One reason for a better memory of the relaxing group at day one, compared to the exercising groups, might be exercise induced arousal and exhaustion. Null effects as well as detrimental effects of physical exercise on cognitive performance were mostly seen in studies with high-intensity exercise protocols, leading to dehydration and exhaustion [59–61]. In the present study, however, participants had the opportunity to drink water during and after the exercise session, making it unlikely that dehydration impaired cognitive processes. Moreover, the intensity of the exercise was individually adjusted to approximately 80% of their maximal heart rate and below 60% for the low-intensity exercise group. Both the high- and low-intensity groups showed worse immediate memory compared to the relaxing group, without any difference between the two making it very unlikely that exhaustion could account for the immediate memory effect. While the exercise groups cycled on a stationary bicycle, the relaxing group sat quietly in a chair. Thus, it is possible that they silently rehearsed the newly learned vocabulary more than the exercise groups which might have improved their immediate memory. However, the relaxing group showed more forgetting after 24 hours compared to the exercise group, suggesting that immediate memory and long-term consolidation were differentially affected by the experimental interventions. Further studies should introduce a task that interferes with memory rehearsal to rule out these effects on memory.

Participants were not asked to recall the vocabulary immediately after learning to avoid any interference effects before exercising. Thus, it is possible that groups differed in the number of words they encoded successfully before exercising. Participants did a word-pseudo-word learning task in the preexperimental screening session which was very similar to the learning task in the main experiment. Groups did not differ with regard to their learning success in this preexperimental task suggesting that there were no baseline differences in learning abilities between groups. However, future studies should include an immediate memory test to control for possible baseline differences in learning success. Furthermore, we argue that in future studies a within-subject design would be more appropriate to assess the effect of exercise on memory to increase the statistical power compared to the current between-subject design. Given the low performance of subjects we expect to find even clearer memory effects by applying a within-subject design.

Serum BDNF was significantly increased after high-intensity exercise, but not after low-intensity exercise. This is in agreement with previous studies reporting an increase of BDNF after acute exercise for high-intensity exercise protocols only [34, 37, 39, 62]. BDNF is an important neurotrophic factor involved in neuronal development and synaptic plasticity. With regard to memory, BDNF is essential for the formation and storage of long-term memories [25]. For instance, results in animals demonstrated that interfering with BDNF expression in the early phase of memory consolidation selectively impairs long-term memory, while recognition memory at the day of acquisition was unaffected [63]. Accordingly, an intracerebroventricular injection of BDNF in rats was found to enhance hippocampus-dependent learning [28]. As acute exercise in humans leads to a transient increase in BDNF, it might be speculated that BDNF mediates the beneficial effects of acute exercise on memory processes. Data on the relationship between an acute exercise induced increase in BDNF and cognitive variables in humans are rare so far. Changes in cognitive tasks like executive functions [34] and attention [38] after acute exercise were found to be unrelated to changes in BDNF. With regard to memory, Winter et al. [20] found a positive correlation between the increase in BDNF after exercise and immediate learning success in a declarative memory task, but no relationship to long-term memory. Griffin et al. [28] reported an increase in BDNF after acute exercise and an increase in a hippocampus-dependent declarative memory task; however, they did not show a correlation between these two variables. For procedural memory, Skriver et al. [64] showed a positive correlation between skill retention up to seven days after learning and serum BDNF, but no relationship to skill acquisition. In the present study, there was a positive correlation between memory consolidation and BDNF increase when analyzing data of all participants. However, this relationship could not be confirmed when only participants of the high-intensity group were included into the analysis. This might be due to a lack of power and smaller variances in this sample.

Furthermore, the less consistent findings on the relationship between an exercise induced increase in BDNF and memory in humans, compared to animal studies, might be due to methodological differences in BDNF assessment. In animals, the local BDNF release in the hippocampus and neocortex is measured, while in human studies BDNF is assessed in the peripheral blood serum. It is disputed whether an increase in central BDNF is accompanied by an increase in serum BDNF. While some results provide evidence for a strong correlation between cortical and serum BDNF [31], other data speak against such a relationship [32]. Nevertheless, BDNF in the serum does not reflect a local measurement of BDNF in specific subregions of the central nervous system, making it harder to detect a correlation with cognitive variables. Thus, a direct link between an acute increase in BDNF and long-term memory consolidation in humans has still to be established.

Psychosocial stress, as well as the application of moderate doses of corticosterone immediately after learning, are known to facilitate memory consolidation [65, 66]. The release of glucocorticoids is increased after physical exercise [67]. Therefore, it has been hypothesized that an exercise induced increase in cortisol might contribute to beneficial effects of a single bout of exercise on memory performance. Saliva cortisol levels were significantly elevated after high-intensity physical exercise in the present study. As in previous studies, we did not observe a cortisol response after low-intensity physical exercise [19, 68]. The increase in cortisol after exercise correlated positively with vocabulary retention after 24 hours suggesting that glucocorticoids release after learning might contribute to the consolidation of newly learned vocabulary. However, the relationship disappeared when only participants of the high-intensity group were considered. Reasons for the lack of a substantial relationship between cortisol and memory could be due to the low variance in this subgroup and the rather small increase in cortisol after exercising in the present study. An inverted U-shaped dose-response function has been described for glucocorticoid effects on memory consolidation [69]. Studies using the cold water test or an aversive psychosocial situation to induce stress reported increases in saliva cortisol around 30% up to 100% [70–73]. The mean increase in cortisol in the present study was 17% in the high-intensity group and, therefore, might have been too low to induce beneficial effects on memory.

Alternatively, the lack of a substantial relationship between neuroendocrinological parameters and memory in the present study might suggest that other mechanisms except an increase in BDNF and cortisol mediate exercise induced changes in cognitive variables. For instance, an exercise induced increase in dopamine and norepinephrine has been linked to better memory performance [20, 22, 64]. Furthermore, acute exercise induces alterations in cerebral blood flow, glucose and lactate levels which modulate learning and memory [64, 74–76].

## 5. Conclusions

The present results provide evidence that high-intensity but not low-intensity physical exercise led to less forgetting of newly learned vocabulary compared to a relaxing group. Furthermore, high-intensity exercise increased the release of BDNF and cortisol in humans. However, it remains an open question whether BDNF and cortisol are mediators of exercise induced benefits on memory. Acute exercise protocols varying the timing of exercise relative to memory encoding and retrieval and the assessment of additional neuroendocrinological parameters might be a promising experimental approach for future studies exploring the factors underlying memory consolidation.

## Supplementary Material

Addional analyses including only women taking contraceptives and men.

## Figures and Tables

**Table 1:** Participants' characteristics and exercise induced changes in heart rate and subjective exhaustion, listed separately for the high-intensity exercise group, low-intensity exercise group, and relaxing group.

	High-intensity exercise group (*N* = 26)	Low-intensity exercise group (*N* = 27)	Relaxing group (*N* = 28)	*p*
Mean age (SD)	21.69 (2.72)	21.63 (2.13)	22.50 (2.22)	0.320^a^
Age range	18–28	19–28	20–29	
Sex (f/m)	13/13	13/14	14/14	0.988^b^
Female: hormonal contraception (y/n)	13/0	10/3	8/6	0.029^b^
Mean BMI (SD)	22.11 (2.00)	21.40 (1.85)	21.55 (1.72)	0.346^a^
Mean self-reported physical activities in MET*∗*h/week (SD)	42.31 (22.23)	45.80 (27.47)	52.94 (26.81)	0.303^a^
Cardiovascular fitness (mean VO_2max_ in mL/min, SD)	2601.74 (707.97)	2611.92 (657.33)	2640.52 (721.99)	0.978^a^
Mean number of foreign languages (SD)	2.35 (1.20)	2.07 (0.55)	2.32 (0.82)	0.467^a^
Playing an instrument (y/n)	21/5	17/10	22/6	0.267^b^
Mean number of correctly recalled words memory pretest (SD)^1^	9.84 (3.94)	9.74 (4.58)	9.64 (4.84)	0.987^a^
Mean heart rate during learning (SD)^2^	74.14 (9.36)	77.31 (9.23)	71.32 (8.88)	0.072^a^
Mean heart rate during intervention (SD)^3^	144.69 (8.48)	98.60 (10.24)	63.15 (8.54)	< 0.001^a^
Mean rating of perceived exhaustion during exercising (SD)	14.54 (1.23)	9.00 (1.80)		< 0.001^c^

^a^ANOVA, ^b^Chi-square test, and ^c^ *t*-test

^1^Missing data for 1 participant of the high-intensity group.

^2^Missing data for 4 participants of the high-intensity group, 1 participant of the low-intensity group, and 3 participants of the relaxing group.

^3^Missing data for 2 participants of the low-intensity group and 1 participant of the relaxing group.
