# Supplementary material for: The Effects of Acute Physical Exercise on Memory, Peripheral BDNF, and Cortisol in Young Adults
Source: Neural Plast. 2016 Jun 29;2016:6860573. doi: 10.1155/2016/6860573 (PMC4942640; doi:10.1155/2016/6860573)
Supplement: Supplementary file 1 — Addional analyses including only women taking contraceptives and men. [file 6860573.f1.pdf]

Supplementary material

Analyses including only women taking contraceptives and men

1. Memory for newly learned vocabulary

Anova results:

Time $F(1, 68) = 3.41$, $p = .069$, $\eta^2 =.048$

Group $F(2, 68) = 2.23$, $p = .115$, $\eta^2 = .062$

Time x Group $F(2, 68) = 4.09$, $p = .021$, $\eta^2 = .107$

Table 1. Descriptive statistics and contrast analysis for memory scores

	high-intensity group<br>N = 26	low-intensity group<br>N = 24	relaxing group<br>N = 21	contrast analysis
Day 1<br>mean (SD)	3.88 (2.66)	3.75 (2.56)	5.67 (3.02)	high-intensity vs. relaxing:<br>*t*(68) = -2.22, *p* = .030<br>low-intensity vs. relaxing:<br>t(68) =- 2.34, p = .022
Day 2<br>mean (SD)	4.08 (2.33)	3.58 (2.50)	4.81 (3.12)	high-intensity vs. relaxing:<br>*t*(68) =- 0.95, *p* = .348<br>low-intensity vs. relaxing:<br>*t*(68) = -1.55, *p* = .125
Difference score day 2 minus day 1<br>mean (SD)	.19 (1.10)	-.17 (1.40)	-.86 (1.28)	high-intensity vs. relaxing:<br>*t*(68) = 2.84, *p* = .006<br>low-intensity vs. relaxing:<br>*t*(68) =1.83, *p* = .071

2. BDNF

Anova results:

Time $F(2, 132) = 5.68$, $p =.004$, $\eta^2 = .079$

Group $F(2, 66) = 1.86$, $p = .163$, $\eta^2 = .053$

Time x Group $F(4, 132) = 16.35$, $p < .001$, $\eta^2 = .331$

Table 2. Descriptive statistics and contrast analysis for the increase in BDNF after exercising

	high-intensity group N = 26	low-intensity group N = 21	relaxing group N = 22	contrast analysis
Difference BDNF t2 minus t0 mean (SD)	2.93 (3.23)	-.72 (2.09)	-1.93 (2.60)	high-intensity vs. relaxing: $t(66) = 6.15$, $p < .001$<br>low-intensity vs. relaxing: $t(66) = 1.45$, $p = .152$

3. Cortisol

Anova results:

Time $F(3, 189) = 15.49$, $p < .001$, $\eta^2 = .197$

Group $F(2, 63) = 0.39$, $p = .676$, $\eta^2 = .012$

Time x Group $F(6, 189) = 5.29$, $p < .001$, $\eta^2 = .144$

Table 3. Descriptive statistics and contrast analysis for the increase in cortisol 20 min after exercising

	high-intensity group N = 26	low-intensity group N = 20	relaxing group N = 20	contrast analysis
Difference cortisol t3 minus t0, mean (SD)	1.08 (4.22)	-1.47 (2.20)	-2.02 (2.00)	high-intensity vs. relaxing: $t(63) = 3.35$, $p = .001$<br>low-intensity vs. relaxing: $t(63) = 0.56$, $p = .578$
